# Supplementary material for: Identification and functional analysis of non-coding regulatory small RNA FenSr3 in Bacillus amyloliquefaciens LPB-18
Source: PeerJ. 2023 May 15;11:e15236. doi: 10.7717/peerj.15236 (PMC10194069; doi:10.7717/peerj.15236)
Supplement: Supplemental Information 4 [file peerj-11-15236-s004.zip › KO/CK-vs-T1_map/map05221.html]

KEGG PATHWAY: Acute myeloid leukemia - Reference pathway


|  |  |
| --- | --- |
| **Acute myeloid leukemia - Reference pathway** |  |

[
Pathway menu
| Organism menu
| Pathway entry
| Show description
| User data mapping
]

Acute myeloid leukemia (AML) is a disease that is characterized by uncontrolled proliferation of clonal neoplastic cells and accumulation in the bone marrow of blasts with an impaired differentiation program. AML accounts for approximately 80% of all adult leukemias and remains the most common cause of leukemia death. Two major types of genetic events have been described that are crucial for leukemic transformation. A proposed necessary first event is disordered cell growth and upregulation of cell survival genes. The most common of these activating events were observed in the RTK Flt3, in N-Ras and K-Ras, in Kit, and sporadically in other RTKs. Alterations in myeloid transcription factors governing hematopoietic differentiation provide second necessary event for leukemogenesis. Transcription factor fusion proteins such as AML-ETO, PML-RARalpha or PLZF-RARalpha block myeloid cell differentiation by repressing target genes. In other cases, the transcription factors themselves are mutated.

|  |  |  |
| --- | --- | --- |
| Reference pathway | 184% 150% 122% 100% 82% 67% 55% | 图片下载 |
